# Lower toxicity and higher efficacy: a study on a novel fully human anti-EGFR antibody

**DOI:** 10.1186/2051-1426-3-S2-P241

**Published:** 2015-11-04

**Authors:** Weiyi Qiu, Shuang Wang

**Affiliations:** 1 Institute of Biotechnology, Academy of Military Medical Sciences, Beijing, People's Republic of China

## 

We generated a novel fully human anti-EGFR antibody showed lower toxicity and higher efficacy in the preclinical, toxicological and pharmacological studies when compared to a commercial drug Cetuximab. In this research, we aimed to understand the probably mechanism.

Firstly, the mAb prefers binding to higher expression of EGFR. Its binding avidity on IgG form of the mAb to EGFR was equivalent to Cetuximab, while the Fab affinity was significantly lower. As a result, Higher EGFR expression tissues like tumor obtained the equivalent efficacy as Erbitux, while normal tissues with lower EGFR expression consequently showed lower toxicity.

Secondly, the new mAb had higher concentration in tumor tissue than Erbitux. The accumulation of antibody in tumor local might induce stronger ADCC effect, and resulted in higher anti-tumor efficacy than Erbitux in a Hu-WBC NOD SCID xenograft mice model.

